# Evaluation of three classification methods of antral follicle count and fertility to the timed artificial insemination in cattle

**DOI:** 10.1590/1984-3143-AR2021-0121

**Published:** 2022-04-20

**Authors:** Fabio Morotti, Suellen Miguez-Gonzalez, Marcela Bortoletto Cerezetti, Marcelo Marcondes Seneda

**Affiliations:** 1 Universidade Estadual de Londrina, Londrina, PR, Brasil; 2 Instituto Nacional de Ciência e Tecnologia para Cadeia Produtiva do Leite, Universidade Estadual de Londrina, Londrina, PR, Brasil

**Keywords:** antral follicle, cattle, insemination, fertility

## Abstract

The controversial data about antral follicle count (AFC) may be partially explained by the different criteria used to determine what is high, intermediate and low AFC. This study evaluated different classification methods for AFC groups, relating them to the conception rate, dominant follicle size and body condition score (BCS) in cows submitted to timed artificial insemination (TAI). Nelore cows (*Bos indicus*; n = 935), received a reproductive program consisting of TAI and natural breeding. Conception rate, BCS and dominant follicle size during TAI were evaluated by three AFC methodologies: i) mean and standard deviation: low (≤ 15 follicles); intermediate (≥ 16 to ≤ 44 follicles) or high (≥ 45 follicles); ii) quartiles: low (≤ 15 follicles); intermediate (≥ 16 to ≤ 39 follicles), or high (≥ 40 follicles); and iii) AFC score: I (low; ≤ 15 follicles); II (intermediate; ≥ 16 to ≤ 30 follicles); III (high; ≥ 31 to ≤ 44 follicles) or IV (very high; ≥ 45 follicles). Data were analyzed by a GLIMMIX and Tukey test or binary logistic regression model (P ≤ 0.05). The conception rate to TAI was influenced (P < 0.05) by AFC in the three methods classification, being the highest conception rate observed in the low AFC group regardless of method utilized: Mean (low 61.73%^a^, intermediate 54.02%^ab^ and high 49.48%^b^), Quartiles (low 61.73%^a^, intermediate 53.59%^ab^ and 51.46%^b^) and Score (I 61.73%^a^, II 54.80%^ab^, III 53.23%^ab^ and IV 49.48%^b^). There were variations (P < 0.05) in the conception rate within the 2.50 to 2.75 BCS range for all AFC classification methods, with the low AFC females presenting the best results, regardless of the method used. Also, females with low AFC showed larger (P < 0.05) diameters of dominant follicles at the TAI regardless of method. The different methodologies used (Mean, Quartile and Score) to AFC classification showed a consistency between the main findings, and we believe that this standardization will facilitate the interpretation of data involving AFC.

## Introduction

The antral follicle count (AFC) has been associated with several parameters linked to fertility in cattle, such as anti-Mullerian hormone dosage (Ireland et al., 2008), progesterone concentration and endometrial thickness (Jimenez-Krassel et al., 2009), oocyte quality (Ireland et al., 2009), superovulation response (Ireland et al., 2007; Silva-Santos et al., 2014), reproductive performance to timed artificial insemination (TAI) (Morotti et al., 2018; Moraes et al., 2019; Lima et al., 2020) and *in vitro* embryo production (IVEP) (Silva-Santos et al., 2014; Santos et al., 2016), pregnancy rate and productive and reproductive longevity (Mossa et al., 2012; Ribeiro et al., 2014; Jimenez-Krassel et al., 2017).

Studies have shown that females with high AFC (≥ 25 follicles) result in better reproductive performance when submitted to reproductive programs involving artificial insemination (Evans et al., 2012; Mossa et al., 2012; Martinez et al., 2016) and *in vivo* and *in vitro* embryo production both *Bos taurus* (Ireland et al., 2007, 2008, 2011; Jimenez-Krassel et al., 2009; Silva-Santos et al., 2014) and *Bos indicus* cattle (Santos et al., 2016; Garcia et al., 2020). However, this reproductive characteristic is still poorly understood and has not been a consensus in the main results among studies (Ireland et al., 2011; Evans et al., 2012; Morotti et al., 2015, 2017). For example, it has been reported that cows with high AFC exhibited better pregnancy rates (Evans et al., 2012; Mossa et al., 2012), but on the other hand studies showed that low AFC resulted in a larger diameter of the ovulatory follicles both *Bos indicus* (Morotti et al., 2018; Lima et al., 2020) and in *Bos taurus* cattle (Bonato et al., 2022), in addition to a higher conception rate (Jimenez-Krassel et al., 2017; Morotti et al., 2018; Moraes et al., 2019; Lima et al., 2020).

Despite several researches, there are still many controversies about the population of antral follicles regardless of the bovine subspecies. Most of the doubts about this topic could be clarified using a standardized methodology regarding the criteria that establish the number of antral follicles for the low, intermediate and high AFC groups in the herd. Some studies established a cutoff without specifically detailing criteria for classification of antral follicle categories (Ireland et al., 2008, 2009; Cushman et al., 2009; Mossa et al., 2012; Jimenez-Krassel et al., 2015, 2017). In some studies, the AFC groups were classified based on the general mean and standard deviation of number of antral follicles from all females evaluated (Santos et al., 2016; Morotti et al., 2018). Another methodology defined the AFC groups by calculating the quartiles of general population of females evaluated, with the low count being defined as AFC ≤ in the 1st quartile and high count being defined as AFC ≥ in the 3rd quartile (Droher et al., 2017; Moraes et al., 2019).

A joint analysis of the main classification methods of the AFC groups would allow an overall assessment of the main findings to identify whether there are important differences in interpretation among the methods. In addition, it would be strategic to suggest a simpler and more standardized way of defining the AFC classifications in a score as it has been used to assess the body condition score (Lowman et al., 1976; Machado et al., 2008; Pfeifer et al., 2017) or to assess reproductive tract in heifers (Andersen et al., 1991).

Thus, these findings indicate that the determination of AFC groups in bovine females can be very variable depending on the methodology employed. In addition, this context reinforces the need for standardization in AFC classification methods, as well as assessing whether there is consistency in reproductive outcomes when different AFC classification methods are used. Therefore, the objectives this study were I) to evaluate the use of different classification methods for AFC groups, relating them to the conception rate, dominant follicle size and body condition score, and II) to compare the pregnancy rate and gestational loss occurring during the breeding season according to AFC. In our study, we hypothesized that the AFC classification methods discussed in this study are consistent in terms of the main reproductive findings, with low AFC females have better fertility than high count in the reproductive program during the breeding season.

## Methods

This study was conducted in accordance with the guidelines of the Ethics Committee on Animal Experimentation at the State University of Londrina, and it was approved under number 5898.2014.76.

### Location, animals and management

This study was conducted during the beef cattle breeding season in two commercial farms in southern Brazil, which were located at 23° 42’ 35” and 51° 45’ 52” (Farm I) and 24° 39’ 01” and 50° 51’ 02” (Farm II). The climate is subtropical and humid (Cfa) according to the Köppen classification with an average temperature of 19.5 °C and an average annual rainfall of 1500 mm. We conducted the experiment with Nelore (*Bos taurus indicus*; n = 935), multiparous females aged between 48 and 120 months, between 40 and 60 days postpartum and with body condition scores (BCS) between 2.5 and 3.5 (scale 1 to 5) (Machado et al., 2008). The conditions for handling the animals were similar between the farms, with the animals being maintained in a continuous grazing system of *Urochloa brizantha* and provided with a mineral mixture diet and water *ad libitum.* Prior to the study, each female was subjected to evaluation of the reproductive tract through palpation and transrectal ultrasound to ensure that only healthy females with no history of reproductive failures were included in the study.

### Hormonal protocol for TAI

**Figure 1:**
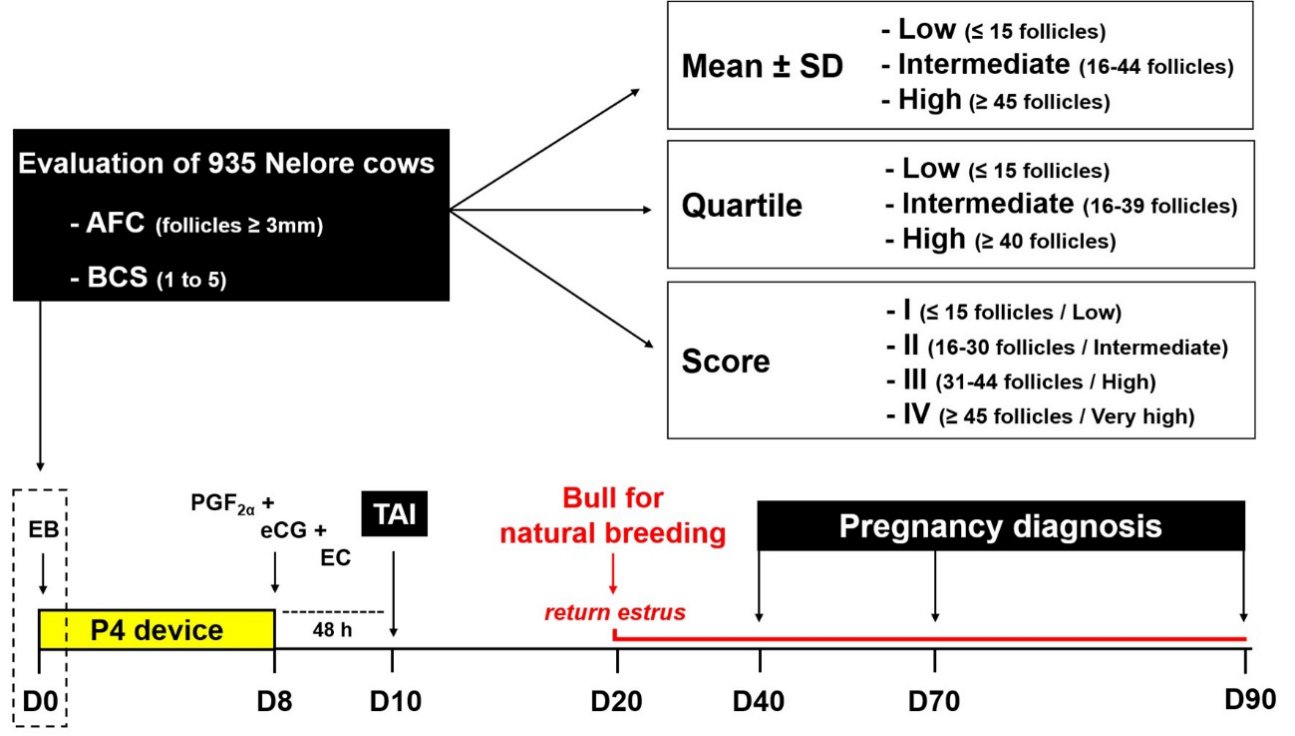
Hormonal treatment for timed artificial insemination (TAI) and experimental design to assess the relationship between antral follicle count (AFC) according to classifications by mean, quartiles and score regarding the pregnancy rate in Nelore cows. P4: progesterone; EB: estradiol benzoate; EC: estradiol cypionate; eCG: equine chorionic gonadotropin; PGF2α: cloprostenol.

All animals were submitted to a conventional ovulation synchronization protocol on a random day of the estrous cycle, designated Day 0 (D0). The protocol consisted of the insertion of an intravaginal progesterone device (P4; DIB®, Zoetis, Brazil) and intramuscular administration (i.m.) of 2 mg of estradiol benzoate (EB; Estrogin®, Farmavet, Brazil). On day 8, the devices were removed, and the animals received applications (i.m.) of 1 mg of estradiol cypionate (EC; ECP®, Pfizer, Brazil), 300 IU equine chorionic gonadotropin (eCG; Novormon®, Syntex AS, Argentina) and 250 μg of cloprostenol (PGF2α; Ciosin®, MSD Animal Health, Brazil), as shown in Figure 1.

All inseminations were performed by a single technician approximately 48 hours after the removal of the intravaginal P4 devices (D10). Conventional frozen-thawed semen was utilized at a temperature of 36 °C for 30 seconds from four bulls with known fertility according to the instructions established by CBRA (2013), and the semen was acquired from a single semen center. Next, after ten days of insemination, Nelore bulls were placed among the batch of cows at a ratio of 1:25 cows with the purpose of performing natural breeding with females that presented estrus. Previously, all bulls had been tested by andrological examinations (CBRA, 2013), and they remained with the females until the end of the breeding season (Day 90).

All females were submitted to ultrasound examination to determine their pregnancy status to the 30 and 60 days after artificial insemination and at the end of the breeding season (Day 90). Pregnancy loss was calculated specifically for inseminated cows through differences in diagnoses of pregnancies between 60 and 90 days.

### Antral follicle count, body condition score and diameter of dominant follicle

To determine the number of antral follicles (AFCs), the ovaries (right and left) of each animal were examined using transrectal ultrasound (Modelo A5V Vet, SonoScape, China) equipped with a linear, rectal 7.5-MHz frequency transducer, and all antral follicles (follicles ≥ 3 mm) were counted as described previously (Burns et al., 2005; Ireland et al., 2008; Morotti et al., 2018) at the beginning of the TAI protocol. In all TAI rounds, the batches of synchronized females were subjected to the same management and feeding practices, and the AFC groups were established only for data analysis.

The evaluation of BCS was performed using visual and tactile evaluation in (D0) by a single evaluator. The evaluation scale was based on the methodology of Machado et al. (2008) on a scale of 1 to 5 (1 - cachectic and 5 - obese) and considering tissue reserves, with emphasis being placed on fat and muscle coverage associated with specific anatomical regions, such as the ribs, spinal and transverse processes of the spine, lumbar vertebrae, iliac and ischial tuberosities, sacrum bone and base of the tail.

To assess the relationship between the diameter of the dominant follicle and the AFC, immediately before the insemination procedure (performed on Day 10), the cows (n = 200) were evaluated by transrectal ultrasound to determine and measure the diameter of the dominant follicle in both ovaries. The average follicular diameter was calculated from two linear cross-sectional measurements of the follicular antrum captured on the ultrasound monitor, with the dominant follicle being the one with an average diameter ≥ 8 mm (Figueiredo et al., 1997).

### Experimental design

**Figure 2:**
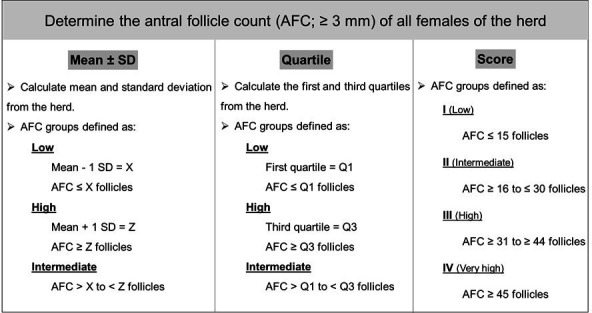
Illustrative scheme of the three methodologies employed (Mean and Standard Deviation, Quartiles and Score) for classification of antral follicle count (AFC; follicles ≥ 3 mm) groups in a single *Bos indicus* cattle subjected to a reproductive program.

To evaluate the different AFC classification methods, all the animals were simultaneously classified by three methodologies as described in Figure 2: I - Mean and Standard Deviation, II - Quartiles, and III - Score. For the classification of the AFC groups according to the mean, the mean number (M) and standard deviation (SD) were calculated from the total antral follicle population of the 935 cows included in this study. Low-count cows were defined based on the population mean of antral follicles (≈ 30 follicles) minus one SD (≈ 15 follicles), wherein the low AFC group had ≤ 15 follicles (n = 243). High counting was defined based on the population mean of antral follicles (≈ 30 follicles) plus one SD (≈ 15 follicles), wherein the high AFC group had ≥ 45 follicles (n = 194). Then, cows within a range of ≥ 16 to ≤ 44 follicles were defined as the intermediate AFC group (n = 498).

Considering the classification of the groups according to the Quartiles, the first quartile (Q1 = 15 follicles, consisting of up to 25% of the animals) and the third quartile (Q3 = 40 follicles, consisting of up to 75% of the animals) were calculated from the total number of antral follicles of all animals. Therefore, females with low AFC were defined as those with lower AFC up to the value of Q1 (≤ 15 follicles, n = 243), cows with high AFC were defined as those with ≥ 40 follicles (n = 274; 25% of the females with the highest AFC) and cows with intermediate AFC were defined as females with follicular counts between Q1 and Q3 (≥ 16 to ≤ 39 follicles, n = 418).

The third classification method includes a suggestion for the present study regarding the classification of animals according to AFC scores. In this method, each female of the evaluated herd was categorized in score I, II, III and IV after considering the sum of the antral follicles of the left and right ovaries. Score I was defined ranging from 1 to 15 follicles (low AFC, n = 243), Score II was established ≥ 16 to ≤ 30 follicles (intermediate AFC, n = 250), Score III was established ≥ 31 to ≤ 44 follicles (high AFC, n = 248) and Score IV was defined to ≥ 45 follicles counted (very high AFC, n = 194). The proposal to classify cows into four groups was to maintain a more homogeneous distribution of the animals in each group, in addition to being one of the first strategies proposed by Burns et al. (2005).

### Statistical analyses

The number of antral follicles and the diameter of the dominant follicles at TAI were analyzed by a GLIMMIX procedure, including the AFC group and farm as the main effects and the BCS as the covariate. In the presence of a significant effect, the means were compared by the Tukey test. The conception rate to TAI was analyzed by a binary logistic regression model, including as main effects the AFC group, the farm and the bull utilized in the insemination. The BCS was included as a covariate of the model. Using the same regression model, gestational loss was analyzed (AFC group as main effect), as well as cumulative pregnancy (AFC group as the main effect and farm and BCS groups as covariates). All data from this study were analyzed using the MINITAB18® statistical software program, version 18.1.1. For significance and interactions, P ≤ 0.05 was utilized, and statistical tendency was determined using a P-value ≤ 0.10. The data are presented as means ± SD or as proportions for the descriptive statistical analysis.

## Results

**Figure 3:**
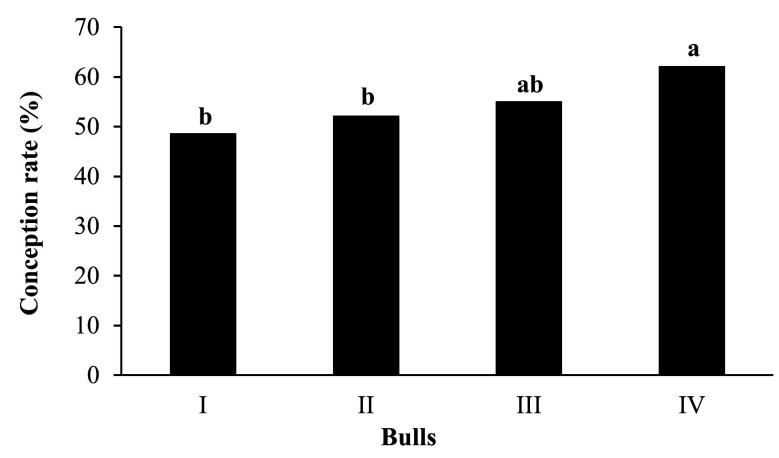
Effect of the bull on the conception rate in beef cows submitted to timed artificial insemination. Bars with different letters (a, b) differ (P = 0.01) among bulls.

The overall pregnancy rates of the study were 55.08% (515/935) at 30 days from TAI and 86.84% (812/935) at the end of the breeding season. In the three methodologies of classifying females according to the number of antral follicles, conception at 30 days was influenced (P < 0.05) by AFC groups and bull but not (P > 0.1) by farm, BCS and interaction AFC*bull (Table 1). Figure 3 shows the conception of the cows according to each bull used in the experiment.

**Table 1:** Conception rate and cumulative pregnancy from Nelore cows submitted to timed artificial insemination (TAI) using different methodologies (Mean and Standard Deviation, Quartiles and Score) for the classification of antral follicle count (AFC) groups.

**Methods for group division**		**Animals**	**AFC**	**Conception from TAI in 30 days**	**Conception from NM in 60 days**	**Gestational loss up to 90 days**	**Cumulative pregnancy**
		(N)	(Mean ± SD)	% (n/N)	% (n/N)	% (n/N)	(TAI + Bull)
							% (n/N)
Mean ± SD	Low (≤ 15 follicles)	243	11.30 ± 2.81 ^c^	61.73 ^a^ (150)	32.92 (80)	7.33 (11/150)	90.12 (219/243)
	Intermediate (16-44 follicles)	498	30.19 ± 7.67 ^b^	54.02 ^ab^ (269)	34.74 (173)	4.46 (12/269)	86.35 (430/498)
	High (≥ 45 follicles)	194	52.77 ± 7.70 ^a^	49.48 ^b^ (96)	38.14 (74)	7.29 (7/96)	84.02 (163/194)
	P-value	-	< 0.0001	0.008	0.13	0.29	0.07
Quartiles	Low (≤ 15 follicles)	243	11.30 ± 2.81 ^c^	61.73 ^a^ (150)	32.92 (80)	7.33 (11/150)	90.12 (219/243)
	Intermediate (16-39 follicles)	418	27.99 ± 6.30 ^b^	53.59 ^ab^ (224)	34.69 (145)	5.36 (12/224)	85.41 (357/418)
	High (≥ 40 follicles)	274	49.53 ± 8.25 ^a^	51.46 ^b^ (141)	37.23 (102)	4.96 (7/141)	86.13 (236/274)
	P-value	-	< 0.0001	0.01	0.12	0.41	0.12
Score	I (≤ 15 follicles)	243	11.30 ± 2.81 ^d^	61.73 ^a^ (150)	32.92 (80)	7.33 (11/150)	90.12 (219/243)
	II (16-30 follicles)	250	23.63 ± 3.93 ^c^	54.80 ^ab^ (137)	32.80 (82)	4.37 (6/137)	85.20 (213/250)
	III (31-44 follicles)	248	36.79 ± 3.94 ^b^	53.23 ^ab^ (132)	36.69 (91)	4.54 (6/132)	86.82 (217/248)
	IV (≥ 45 follicles)	194	52.76 ± 7.70 ^a^	49.48 ^b^ (96)	38.14 (74)	7.29 (7/96)	84.02 (163/194)
	P-value	-	< 0.0001	0.02	0.17	0.48	0.15
Total/Mean		935	29.97 ± 15.63	55.08 (515/935)	34.97 (327)	5.82 (30/515)	86.84 (812/935)

Values followed by lower case letters (a, b) and within the same column differ statistically (P < 0.05) between the AFC groups. NM: Natural mating.

The conception rate for natural breeding (return estrus), the pregnancy rate accumulated at the end of the breeding season and the gestational loss did not change (P > 0.05) due to the AFC group, the farm or the BCS, regardless of the methodology for AFC classification used (Table 1).

The diameter of the dominant follicle at the time of TAI differed (P < 0.05) among the AFC groups, regardless of the methodology used (mean, quartiles and score) for the classification of females (Table 2), and in general groups with low AFC resulted in larger follicle diameter at TAI (P < 0.05) when compared to the high AFC group.

**Table 2:** Diameters of dominant follicles (mm) in Nelore cows (n = 200) with different antral follicle counts (AFCs) submitted to timed artificial insemination (TAI).

**Methods for group division**		**Animals (N)**	**Diameter of dominant follicle (Mean ± SD)**
Mean ± SD	Low (≤ 15 follicles)	61	11.83 ± 2.39 ^a^
	Intermediate (16-44 follicles)	104	11.25 ± 2.62 ^ab^
	High (≥ 45 follicles)	35	10.34 ± 2.46 ^b^
	P-value	200	0.03
Quartiles	Low (≤ 15 follicles)	61	11.83 ± 2.39 ^a^
	Intermediate (16-39 follicles)	81	11.34 ± 2.62 ^ab^
	High (≥ 40 follicles)	58	10.45 ± 2.50 ^b^
	P-value	200	0.01
Score	I (≤ 15 follicles)	61	11.83 ± 2.39 ^ab^
	II (16-30 follicles)	51	12.03 ± 2.46 ^a^
	III (31-44 follicles)	53	10.55 ± 2.67 ^bc^
	IV (≥ 45 follicles)	35	10.34 ± 2.46 ^c^
	P-value	200	0.002

Values followed by lower case letters (a, b), within the same column and to same methods differ statistically (P < 0.05) between groups with different follicular counts.

There were no differences (P > 0.1) in the conception rate between different BCS ranges (2.50 to 2.75 vs. 3.00 to 3.50). However, cows with 2.50 to 2.75 BCS resulted in significant variations (P < 0.05) in the conception rate and in general, the high count showed the lower conception rates compared to low count, regardless of the AFC classification methods employed in the study (Table 3).

**Table 3:** Conception rate to timed artificial insemination (TAI) from Nelore cows with different antral follicle counts (AFCs), classified by different methodologies (Mean and Standard Deviation, Quartiles and Score), according to the range of body condition scores (BCSs).

**Methods for group division**		**BCS range**		**Overall conception % (n/N)**
		2.5 to 2.75% (n/N)	3.0 to 3.50% (n/N)	
Mean ± SD	Low (≤ 15 follicles)	62.37 ^a^ (116/186)	59.65 (34/57)	61.73 ^a^ (150/243)
	Intermediate (16-44 follicles)	53.64 ^b^ (184/343)	54.84 (85/155)	54.02 ^ab^ (269/498)
	High (≥ 45 follicles)	46.46 ^b^ (59/127)	52.22 (37/67)	49.48 ^b^ (96/194)
	P-value	0.008	0.74	0.01
Quartiles	Low (≤ 15 follicles)	62.37 ^a^ (116/186)	59.65 (34/57)	61.73 ^a^ (150/243)
	Intermediate (16-39 follicles)	53.58 ^b^ (157/293)	53.60 (67/125)	53.59 ^ab^ (224/418)
	High (≥ 40 follicles)	48.59 ^b^ (86/177)	56.70 (55/97)	51.46 ^b^ (141/274)
	P-value	0.01	0.66	0.02
Score	I (≤ 15 follicles)	62.37 ^a^ (116/186)	59.65 (34/57)	61.73 ^a^ (150/243)
	II (16-30 follicles)	55.87 ^ab^ (100/179)	52.11 (37/71)	54.80 ^ab^ (137/250)
	III (31-44 follicles)	51.22 ^b^ (84/164)	57.14 (48/84)	53.23 ^ab^ (132/248)
	IV (≥ 45 follicles)	46.46 ^b^ (59/127)	55.22 (37/67)	49.48 ^b^ (96/194)
	P-value	0.01	0.84	0.02
Total/Mean		54.72 (359/656)	55.91 (156/279)	55.08 (515/935)

Values followed by lower case letters (a, b), within the same column and to same methods differ statistically (P < 0.05) between the different body condition scores (BCS).

## Discussion

The present study examined three distinct methodologies for AFC classification (Mean, Quartiles, and Scores) on the conception rate to TAI, related them with possible influence of BCS and diameter of the dominant follicle in the insemination time. Based on our results, we suggest the use of classification by score, as it did not result in important differences in relation to other AFC classification methods, in addition to representing a simpler and more practical application strategy. In addition, in Nelore cattle, this study is the first to investigate the relationship between AFC and pregnancy rate after natural breeding (breeding with bull on return of estrus after TAI), with gestational loss and with the cumulative pregnancy rate at the end of the breeding season.

The results obtained in this study show that regardless of the methodology used, there is a consensus regarding the main findings among AFC groups and fertility after TAI. This finding is highly relevant because it minimizes the concern regarding the controversial aspects of AFC and fertility being attributed to the different methodologies employed (Ireland et al., 2011; Morotti et al., 2015, 2017; Zangirolamo et al., 2018).

We demonstrated that independent of the methodologies adopted for AFC classification, the relationship between the conception rate and AFC groups seems to follow the same standard. Additionally, the present study proposes that the classification of the AFC groups be presented in the form of a score due to objectivity in application. This strategy would result in a standardization among studies, researchers, and field technicians, favoring data analysis and increasing the reliability of findings. In addition, this strategy would facilitate the practical application of this reproductive characteristic in commercial reproductive programs, as has been proposed with BCS by Lowman et al. (1976), Machado et al. (2008) and Ayres et al. (2009). In yet another aspect, it is worth noting that the AFC classification by mean and standard deviation and quartiles may vary in function of the global population of antral follicles in the herd, and therefore the cutoff points established in this study may not be suitable to be applied in other studies, needing to extract these values from each herd. When using the AFC classification in score, the proposal is that regardless of the subspecies, race or aptitude of the herd analyzed, each female is categorized within one of the scores proposed in this study.

Regardless of the AFC classification methodology that is employed (Mean, Quartiles or Score), Nelore females with a low number of antral follicles exhibited the higher conception rate after receiving the TAI protocol when compared to the high AFC group. Although the bull demonstrated an effect on the pregnancy rate at TAI, no interaction with AFC was observed, which supports that these effects occurred in isolation in the present study. In Nelore females, it has been demonstrated that a low AFC results in a higher pregnancy rate at TAI (Morotti et al., 2018; Moraes et al., 2019; Lima et al., 2020).

The conception rate after natural breeding, gestational loss and pregnancy rate at the end of the breeding season did not differ among the AFC groups, regardless of the classification methodology that was employed. Although the hypothesis has not been fully supported, it is worth noting that increased conception at the beginning of the reproductive season has numerous advantages and is one of the goals of reproductive programs (Marques et al., 2015; Baruselli et al., 2018; Vaz et al., 2020). Thus, the AFC classification can be used as a reproductive strategy to increase the proportion of pregnancy insemination in the herd at the beginning of the breeding season.

Regarding the size of the dominant follicle, low-count females also exhibited larger diameters in relation to high-count animals, regardless of the methodology employed. This result is in agreement with that reported by Morotti et al. (2018) and Lima et al. (2020). The larger diameter of the dominant follicle has been considered an important feature linked to the fertility of *Bos indicus* females in TAI. This importance is due to the positive relationship of the follicular diameter at insemination and the greater ovulatory potential, the greater size of the corpus luteum and the concentration of progesterone, as well as a higher probability of pregnancy (Meneghetti et al., 2009; Sá et al., 2010; Pfeifer et al., 2012, 2015). Thus, it is suggested that AFC may be a tool to increase the pregnancy rate in the herd, improving reproductive efficiency.

Considering that AFC is a highly repeatable characteristic, that its variation is associated with reproductive performance and that there is an advantage of low AFC when TAI is performed, strategies for enhancing the reproductive performance of *Bos indicus* cattle can be suggested. For example, it could be suggested that females of low and medium AFC be directed to the first breeding programs of insemination within the breeding season, favoring a more efficient use of the TAI tool. Furthermore, considering that females of low AFC have larger diameters of dominant follicles, it would be possible to suggest a more costly semen targeting in these animals to obtain better economic viability and efficiency in the use of semen, similar to that proposed by Silveira et al. (2018) and Rosa et al. (2019).

In the present study, there was a relationship between AFC and BCS, and although cows with BCS > 3.00 did not show variation in TAI conception rates according to AFC groups, the range from 2.50 to 2.75 showed significant variation, with females with low AFC presenting a higher conception rate compared to the high count, regardless of the classification method used. A relationship between AFC, BCS and TAI performance was also investigated by Moraes et al. (2019); however, in contrast to the results of the present study, the authors showed that this relationship occurred for females with BCS > 3.00, which was also favorable for the low AFC group in relation to high count, as indicated by the present study. Research elucidating this relationship between AFC and BCS has not been reported. In dairy cattle, for example, it is believed that this relationship may be related to a greater susceptibility of females with high AFC to productive and health challenges, as observed by Jimenez-Krassel et al. (2017). In addition, in both dairy and beef cattle, a possible interaction with factors related to metabolic and hormonal disorders in the postpartum period is noted, as well as factors related to nutritional management and energy balance in the pre- and postpartum period (Soca et al., 2014; Meteer et al., 2015). Therefore, the relationship between metabolic status and ovarian activity is possibly affected by the ovarian follicular population.

Finally, the present study observed that the classification of AFC through the three methodologies (Mean, Quartiles and Score) appeared to be reliable without variations among the main investigated results. The findings of this study may help to answer some of the questions that have been raised. In addition, the possibility of employing the AFC classification through the scoring methodology makes the process of implementing this reproductive characteristic simpler, more practical and more reliable, facilitating the use of AFC as a reproductive tool, similar to the way that BCS is used. Additionally, the AFC classification proved to be a strategic tool because it shows that in the first 40 days of the breeding season using TAI, the higher proportion of pregnancies was observed in females with lower follicular counts compared to those with high AFC. Although the relationship between AFC and BCS has not been elucidated to date, females with lower BCS are more likely to exhibit pregnancy success, and a larger diameter of the dominant follicle at the time of insemination is associated with the best results of pregnancy following TAI in low-count *Bos indicus* animals.

## Conclusion

The different methodologies employed (Mean, Quartile and Score) were presented as viable strategies in the classification of *Bos indicus* females according to the number of antral follicles. The methods demonstrated similarities among the main findings, indicating reliability regardless of the methodology that was utilized; therefore, for practical reasons, this study suggests the use of the score methodology. The rate of pregnancy due to the natural breeding, the gestational loss and the pregnancy rate at the end of the breeding season were not related to the AFC. However, females with low AFC showed larger diameters of dominant follicles at the time of insemination and exhibited a better pregnancy rate at the beginning of the breeding season compared to those with high AFC. In addition, AFC showed a relationship with lower body condition score in females.
